# Regional responsibility and coordination of appropriate inpatient care capacities for patients with COVID-19 – the German DISPENSE model

**DOI:** 10.1371/journal.pone.0262491

**Published:** 2022-01-27

**Authors:** Benedict J. Lünsmann, Katja Polotzek, Christian Kleber, Richard Gebler, Veronika Bierbaum, Felix Walther, Fabian Baum, Kathleen Juncken, Christoph Forkert, Toni Lange, Hanns-Christoph Held, Andreas Mogwitz, Robin R. Weidemann, Martin Sedlmayr, Nicole Lakowa, Sebastian N. Stehr, Michael Albrecht, Jens Karschau, Jochen Schmitt

**Affiliations:** 1 Center for Evidence-based Healthcare, University Hospital Dresden and Medical Faculty Carl Gustav Carus, TU Dresden, Dresden, Germany; 2 University Center of Orthopaedic, Trauma and Plastic Surgery, University Hospital Carl Gustav Carus, Dresden, Germany; 3 Institute for Medical Informatics and Biometry, University Hospital Dresden and Medical Faculty Carl Gustav Carus, TU Dresden, Dresden, Germany; 4 Quality and Medical Risk Management, University Hospital Carl Gustav Carus Dresden, Dresden, Germany; 5 Clinic for Infectious Diseases and Tropical Medicine, Klinikum Chemnitz, Chemnitz, Germany; 6 Department of Anesthesia and Critical Care Medicine, Leipzig University Hospital, Leipzig, Germany; 7 University Hospital Carl Gustav Carus Dresden, Dresden, Germany; Kaohsuing Medical University Hospital, TAIWAN

## Abstract

As of late 2019, the COVID-19 pandemic has been a challenge to health care systems worldwide. Rapidly rising local COVID-19 incidence rates, result in demand for high hospital and intensive care bed capacities on short notice. A detailed up-to-date regional surveillance of the dynamics of the pandemic, precise prediction of required inpatient capacities of care as well as a centralized coordination of the distribution of regional patient fluxes is needed to ensure optimal patient care. In March 2020, the German federal state of Saxony established three COVID-19 coordination centers located at each of its maximum care hospitals, namely the University Hospitals Dresden and Leipzig and the hospital Chemnitz. Each center has coordinated inpatient care facilities for the three regions East, Northwest and Southwest Saxony with 36, 18 and 29 hospital sites, respectively. Fed by daily data flows from local public health authorities capturing the dynamics of the pandemic as well as daily reports on regional inpatient care capacities, we established the information and prognosis tool DISPENSE. It provides a regional overview of the current pandemic situation combined with daily prognoses for up to seven days as well as outlooks for up to 14 days of bed requirements. The prognosis precision varies from 21% and 38% to 12% and 15% relative errors in normal ward and ICU bed demand, respectively, depending on the considered time period. The deployment of DISPENSE has had a major positive impact to stay alert for the second wave of the COVID-19 pandemic and to allocate resources as needed. The application of a mathematical model to forecast required bed capacities enabled concerted actions for patient allocation and strategic planning. The ad-hoc implementation of these tools substantiates the need of a detailed data basis that enables appropriate responses, both on regional scales in terms of clinic resource planning and on larger scales concerning political reactions to pandemic situations.

## Introduction

The COVID-19 pandemic affects societies worldwide. It began spreading across Europe and North America in early 2020. From the very beginning, the COVID-19 pandemic has been a significant challenge to health care systems worldwide. The health care capacities of many countries reached their limits in regions with particularly high SARS-CoV-2 infection numbers due to a fast acceleration of the infection dynamics [1–4]. The activity and regional dynamic of COVID-19 is subject to substantial regional and temporal variations. As of February 2021, Germany had experienced two waves of the COVID-19 pandemic. These two waves, in spring 2020 and in winter 2020/2021, differed from one another in regard to absolute numbers, regions most affected, and risk factor distribution for severe COVID-19 disease [5]. The challenge for the healthcare system under such pandemic pressure is to provide as many inpatient and intensive care capacities for COVID-19 patients as needed and, at the same time, to provide appropriate care for all other patients. In Germany, there were many regions where the first wave of the pandemic had only a moderate impact, such that only few capacities were needed to provide appropriate care for COVID-19 patients. However, hospital care for the majority of chronic diseases had been significantly reduced in all parts of Germany, potentially leading to undersupply for many patients, as a result of measures taken to be prepared for a potential urgent need for large COVID-19 capacities of care [6]. In hospital care, the aim is thus to prevent situations of potential undersupply, while at the same time staying responsive to the quickly changing requirements of COVID-19 capacities. Adequate planning of such appropriate hospital capacities for COVID-19 and, as a consequence, the best capacities possible for all other patients requires regional transparency, central coordination, cooperation between inpatient and outpatient care as well as data exchange between public health institutions and clinical care providers. Currently, the German healthcare system does not meet these requirements for a coordinated regional response of the healthcare system to the pandemic situation. In Germany, as in most other healthcare systems, hospitals rarely interact directly. There is neither a flow of structured data nor is there regional coordination between different hospitals or between hospitals and public health authorities. Instead, hospitals act as individual players on the market, which poses significant limitations in terms of regional pandemic preparedness [7]. Comparable conditions in Bergamo and Vó (Italy) lead to a struggle with high rates of infections and deaths during the first COVID-19 wave in spring 2020. This gives rise to the suspicion that insufficiently informed regional coordination of COVID-19 care may have contributed to the overburdening of hospitals in addition to the complex medical attention required to care for individual patients. Under non-pandemic circumstances, emergency services carry acute patients to the nearest hospital. However, during COVID-19, mass outbreaks in, for example, nursing homes arguably would lead to large numbers of patients admitted to a single hospital under such an allocation strategy. The affected hospital would then have little time, facilities or personnel capacities to appropriately admit and isolate the overflow of patients in a structured manner. Even if the hospital were to refuse admission of a patient, emergency services would take that patient to the next nearest hospital. In a pandemic, this strategy leads to knock-on and spill-over effects at the next highest level [1, 8]. Based on the conceptual framework of value-based healthcare [9], we established a different strategy, namely a regional model of care that enables a collective response to real-time pandemic activity, provides just as many capacities for COVID-19 patients as needed and leaves the remaining capacity for all non COVID-19 patients, thus aiming for the best possible care for both patient groups. A crucial part of this model of care is the estimation of near-future hospital bed demands via mathematical modeling.

The aims of this paper are as follows:

1. Description of the regional Saxonian model of care for pandemic response
2. Presentation and validation of our mathematical model for predictions of regional demands of bed capacities.

## Materials and methods

### Non-pharmaceutical interventions in Saxony, Germany, during the second wave

Germany is a federal republic with strict separation of power and responsibilities between the federal government and its 16 states, such that non-pharmaceutical interventions (NPIs) and most regulations concerning COVID-19 are established at the state level and are only coordinated by the federal government. Thus, in principle every state has its one set of regulations that depends on the local pandemic situation and respective political measures taken on the state level. During the second wave of the pandemic between October 1 and December 24, 2020, the state of Saxony readjusted its regulations five times tightening restriction with each subsequent set of regulations to counteract the increasing number of cases [10–15]. Among these five adjustments, the introduction of a moderate shutdown on November 2, 2020, and the implementation of an extended shutdown on December 13, 2020, mark significant changes in policies.

In October 2020, light restrictions introduced during the first wave early 2020 were still in place, e.g., a ban of public gatherings of more than two households plus family members and ten additional people as well as mandatory masks in shops and public transport, with additional regional restrictions depending on the number of new cases per week and the overall population.

On November 2, 2020, these counter measures became significantly stricter. Shops, bars, restaurants, sport facilities and most public meeting places were closed, non-private public events were generally restricted in their number of participants and the allowed number of simultaneous private contacts was reduced. Schools were mostly unaffected by these regulations.

This changed on December 14, 2020, when the state of Saxony entered into an extended shutdown, which further reduced the allowed number of private and public contacts and facilitated the closing of schools and essential educational institutions. These were thereupon closed.

Both shutdowns, moderate and extended, had a significant effect on the infection dynamic and bed occupancy rates but did not prevent the number of cases from increasing.

### Regional setting in Saxony

Saxony is the tenth largest of Germany’s sixteen states (area of 18,413 km^2^) and with more than 4 million inhabitants the sixth most populous state [16]. Saxony is divided into thirteen counties with three larger cities (each with approximately 250,000 to 590,000 inhabitants). The NUTS 2 (Nomenclature of Territorial Units for Statistics) divides Saxony into three regions, East, Northwest and Southwest, which contain clusters of 36, 18 and 29 acute hospital locations, respectively. For reasons of simplicity, we will refer to those three regions as “East”, “North” and “West”. In addition, as the focus of this paper is on the facilities rather than the organizations, we will refer to “locations of hospitals” as “hospitals” throughout. Beyond these clinics, other medical facilities such as rehabiliation centers provide inpatient care. In case of high demand during the pandemic, such facilities are capable of temporarily providing emergency capacities.

Due to five rescue coordination centers in Saxony for regional patient distribution the establishment of central COVID-19 coordination centers (clinic coordination centers) was necessary to ensure a structured distribution of patients. These clinic coordination centers are located at three hospitals of maximum care, which are the large university hospitals in Dresden (East), Leipzig (North) and the clinic of Chemnitz (West). The distribution of COVID-19 patients based on severity of disease and needed treatment (normal ward or intensive care) is organized by a graduated scheme:

- Level I: inside of the three clusters East, North and West Saxony
- Level II: inside Saxony between different clusters
- Level III: nationwide in Germany [17]

On a daily basis, the individual hospitals report their actual occupancy of COVID-19 beds as well as free beds to the clinic coordination center of their cluster. Their coordination center then provides this information to be fed into the DISPENSE database (see below for abbreviation). In return, the DISPENSE tool provides a daily report on free and occupied clinic beds for each cluster as well as a prognosis and in this way enables the clinic coordination centers to allocate COVID-19 patients to clinics with free treatment capacities. Video conferences with all heads of each of the clinics provide a planning, information dispersal and mutual discussion platform. These conferences were held up to twice a week during November and December 2020.

On another scale of surveillance, thirteen local health departments oversee and monitor SARS-CoV-2 outbreaks. They all report their information to the State Health and Veterinary Research Institute (“Sächsische Landesuntersuchungsanstalt”), a subordinate authority of the Saxon State Ministry for Social Affairs and Social Cohesion at the next higher state-level of organization, which in turn relays this data to the DISPENSE database.

The data, e.g. information about age, gender, and date of death of Saxonian COVID-19 patients, is also reported to the Robert-Koch-Institute at the federal state-level. Note that in contrast to the DISPENSE tool, which has been established as a local solution as part of a sudden demand during the pandemic, this structure of reporting from the local authorities to the federal state level is part of the German public health service.

### East Saxony and DISPENSE

In March 2020, the German federal state of Saxony, in the course of a general ruling, assigned the regional responsibility for the provision of appropriate inpatient care facilities for COVID-19 patients in East Saxony to the University Hospital Carl Gustav Carus in Dresden (University Hospital Dresden hereafter) where the clinic coordination center is situated alongside the 35 hospital. On these legislative grounds, the DISPENSE (short for: *COVID-19*: *Dresdner Informations- und Prognosetool für Erkrankungsverlauf und Bettenauslastung in Sachsen*, in English: *COVID-19*: *Dresden’s information and prognosis tool for the disease course and hospital bed demand*) model of care was initiated by the University Hospital Dresden together with its 35 partner hospitals and the local public health institutions. The acronym comprises the idea of an online tool including infection surveillance and prognosis of bed capacity requirements in Saxon regions. The clusters East, North and West Saxony had agreed on and established a collection of data for bed occupancies. Those data were subsequently integrated into the DISPENSE tool. The project was undertaken in accordance with the GCP and data protection legislation. The institutional review board of the TU Dresden approved the study protocol (EK number: 235062020). Note that as no patient data was used, no consent was required.

Central elements of the **DISPENSE model of care** are:

- Formalized regional network of all hospitals including acute care and rehabilitation care
- Central clinic coordination center
- Integration of regional public health authorities and pandemic surveillance institutions
- Standardized collection and reporting of relevant data needed for pandemic management
- Near-future prediction of regional inpatient and intensive care demands for COVID-19 patients

The **DISPENSE Tool** provides standardized, automated, daily reporting of relevant data for regional pandemic management and includes a module for prediction of hospital and intensive care unit (ICU) capacity needs for the upcoming 2 weeks. It consists of individual components to handle data automatically at various stages of the process of extraction, transfer and loading (ETL).

**Fig 1:**
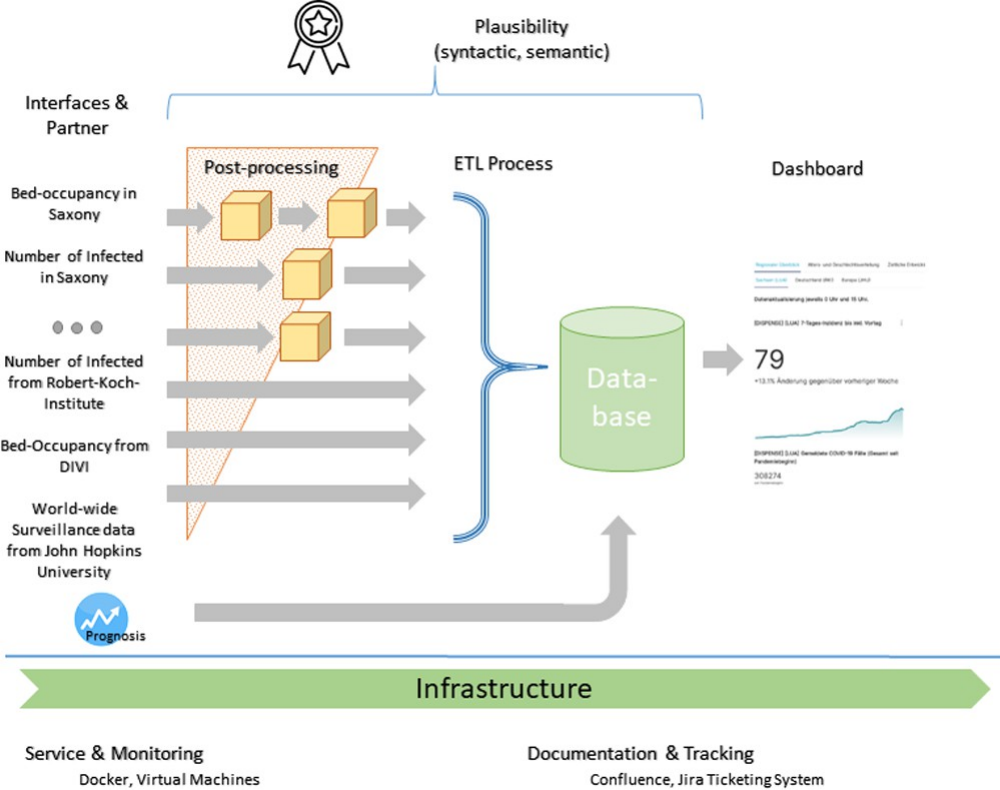
Data flow of the DISPENSE tool. The DISPENSE tool integrates data transfer, load and extraction into a one-stop-shop solution. In the back-end a database holds all relevant data for a visual data dashboard at the front-end that displays the requested information to the user. All modules run in a container-based infrastructure in components shown at the bottom of the scheme.

The extraction of data from several heterogeneous sources is funneled through a custom-made system of post-processing steps and culminates in a complementary database as is shown in Fig 1. Depending on the particular source, data capture is from structured open records (e.g. Robert-Koch Institute, Johns-Hopkins University) or is based on custom-made solutions such as Excel spreadsheets or web-based self-reporting systems (e.g. survey systems). All data is first saved in a data lake from which relevant information is distilled and stored in a database. In detail, information is captured at several levels to monitor:

- The COVID-19 infection spread on
  ◦ the local and communal level (received from the State Health and Veterinary Research Institute)
  ◦ the regional Saxony-wide level (statewide information on a fast track data download to avoid reporting delays to the Robert-Koch-Institute)
  ◦ the federal Germany-wide level (data is sourced from an open data source provided by the Robert-Koch-Institute) [18]
  ◦ the European level (data is sourced from John Hopkins University data set) [19]
- The normal ward and ICU ward bed occupancy from all three Saxon hospital clusters centrally managed by the University Hospital Dresden (Cluster East with 36 acute hospital sites), University Hospital Leipzig (Cluster North with 18 acute hospital sites), and the hospital Chemnitz (Cluster West with 29 acute hospital sites)

**Fig 2:**
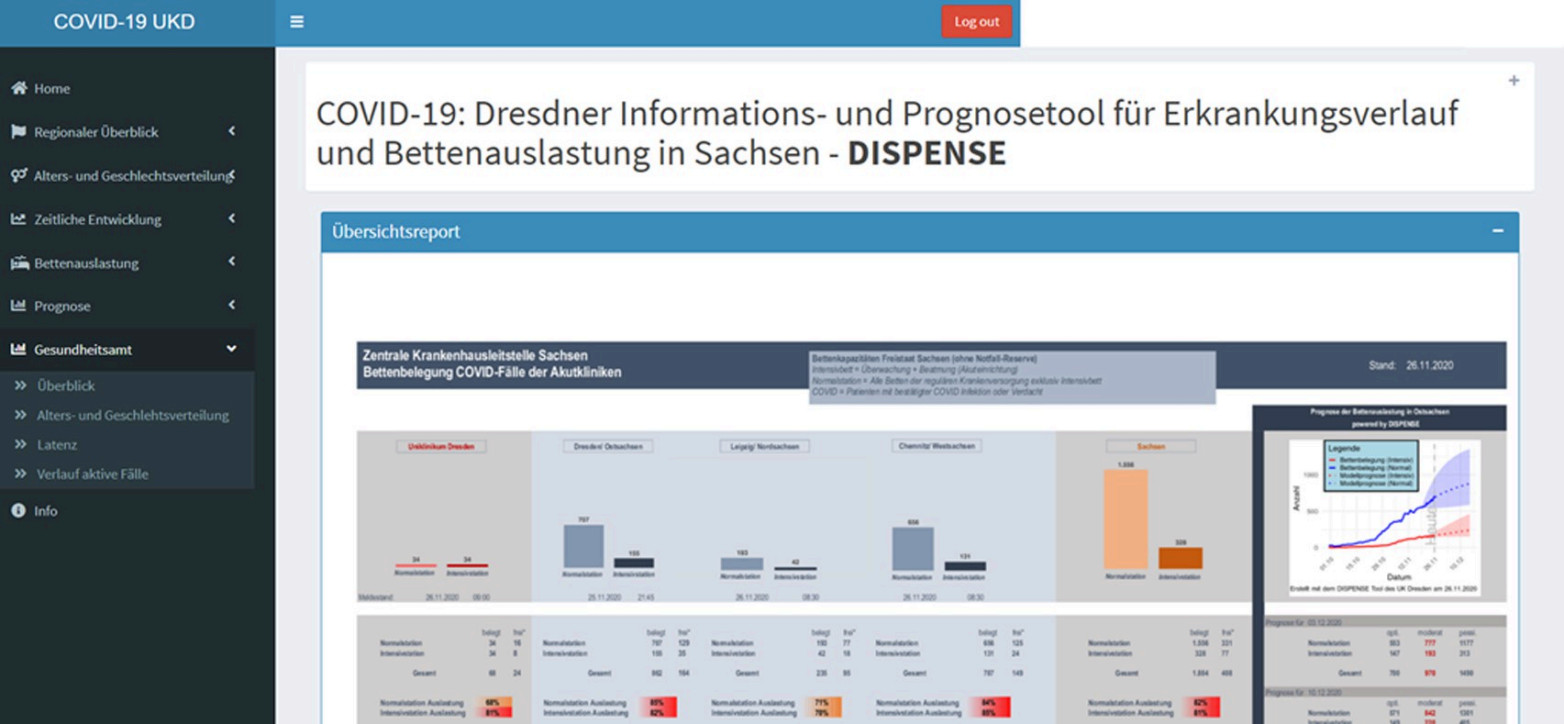
Screenshot of the shiny front-end of the DISPENSE tool. Welcome page displaying key data, performance indicators and plots to decision makers. The left-hand bar enables the user to dive deeper into a more detailed data analysis and prognosis.

The front-end, a tool implemented in R Shiny displays plots and key figures to the end-users of the crisis management and infection monitoring teams, i.e. decision makers at regional hospitals, health administration authorities, and ministries. For this purpose, the most suited dataset is utilized for each visualization, e.g. state-provided data for Saxony and the John Hopkins University data set for a European overview. The presentation is both in electronic form of an internet dashboard (see Fig 2) and materialized as a printable PDF report.

The dynamic mathematical model to predict near future bed occupancies in Saxony is driven by the most recent and accurate data sources for incidences and current bed occupancies in Saxony, i.e. the state provided infection data and detailed bed occupancy data provided by the three clusters. A prototype of the mathematical model for prognosis was set up and used by decision makers in East Saxony as of early April 2020. The prognosis was implemented as a fully automatized version for all three clusters in a development phase from summer 2020.

The DISPENSE tool is regularly used by the central clinic coordination centers and regional clinics for strategic decisions in pandemic management for the graduated distribution scheme, creation of additional treatment capacity by the reduction of elective medical treatment and data reports in video conferences of the cluster clinics. The reliable prediction of COVID-19 patient numbers on a two-week horizon via DISPENSE prognoses allows for proactive and tailored disaster management.

### Mathematical prognoses for hospital bed planning

**Fig 3:**
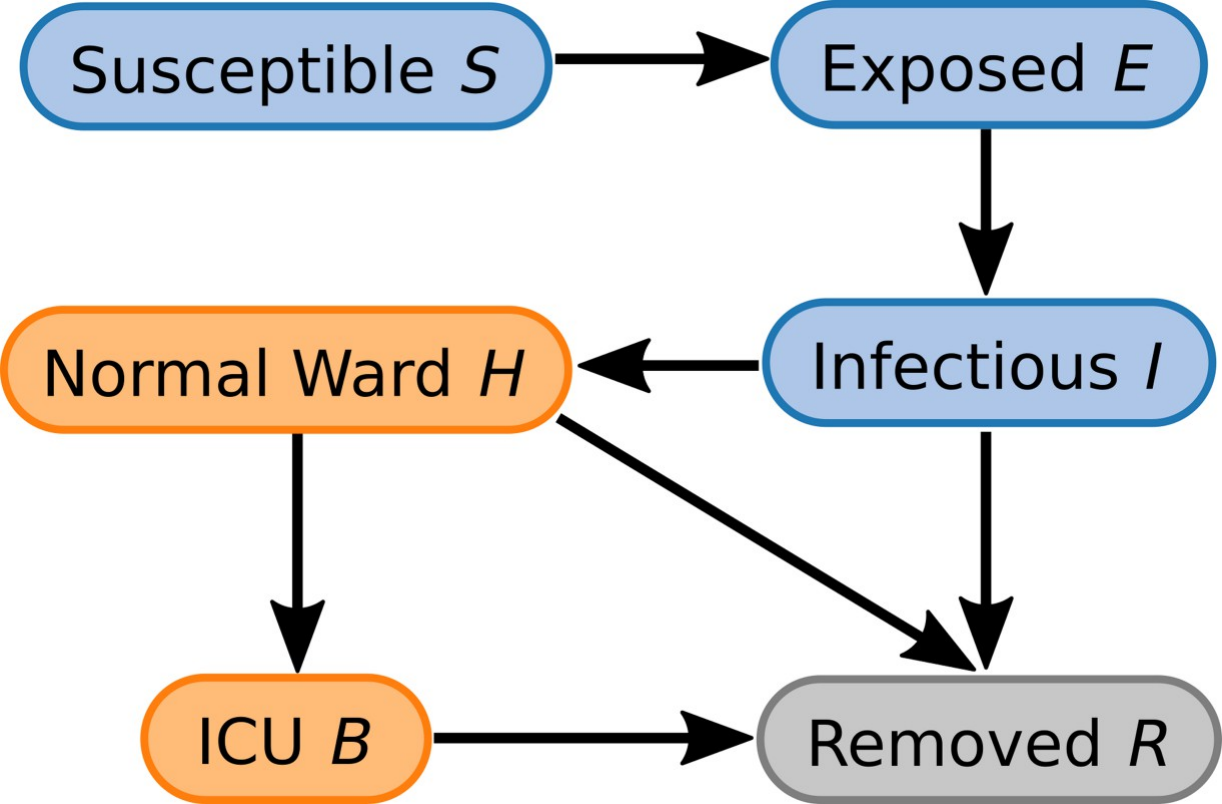
Compartment structure of the employed SEIR model. The traditional model is extended by the normal ward and ICU compartments.

In order to estimate the course of the pandemic at the population and health care level we used a variant from the Susceptible-Exposed-Infected-Removed (SEIR) model class [20–24]. Fig 3 shows the compartments of our model and their interconnections; the full model equations can be found in the S1 File.

Like in classical SEIR-models, susceptible individuals in the compartment (S) are transferred to the compartment (E) whenever they are exposed and infected by contact with an infectious individual in compartment (I). Exposed individuals in compartment (E) are infected but incapable of infecting others as, for example, their viral load is still too low. They then transition to the compartment (I) after a pathogen specific waiting time and become infectious themselves.

Unlike the number of infectious individuals in compartment (I), the amount of people in compartment (E) cannot be measured robustly with standard PCR or rapid antigen test.

During the course of the infection, the infectious person then leaves this state by transitioning to the removed (R) or normal ward state (H). Please note that the removed state (R) comprises all people who have either recovered from the disease or passed away as a result from or during the disease. Whether a person is removed from or enters into the normal ward state depends on a specific probability. This probability encapsulates several risk factors to enter the hospitalized stage, e.g. age, comorbidities, or overall fitness. As these factors are both complex and/or not known, they do not enter into the model explicitly, but are contained in the model parametrization.

The hospitalized patients then transition from the normal ward (H) either to the removed state (R) or to the ICU state (B). The choice of this route is random with probabilities that again depend on the aforementioned risk factors. Since the direct admission form infectious (I) to ICU ward (B) played a secondary and negligible role in Saxony for the largest part of the pandemic and parameter estimation for this transition proved impossible with the provided data set, we decided against modeling this transition.

Thus, parameters of this model are the transition rates between compartments, transition probabilities and the overall infection parameter R0. Table 1 summarizes the values of all fixed parameters. Note that values for the transition probabilities and the overall infection parameter R0 are not fixed. Instead, they serve to adjust the model dynamics to changes during the course of the pandemic. Thus, the free parameters are the probability to transition from the infectious state (I) to the normal ward state (H), the probability to transition from the normal ward state (H) to the ICU (B), and the R0 value. The latter describes the spreading dynamics within the total population while the former two describe the risk factors to enter a hospitalized state or escalate to a state requiring intensive care.

**Table 1:** Overview of model parameters.

Parameter	Parameter Value	Meaning and Interpretation
r_EI_	1/3 per day	mean rate from the exposed to the infectious state
r_IR_	1/14 per day	mean rate from infectious to the removed state
r_IH_	1/4 per day	mean rate to transition from the infectious to the hospitalized state at the normal ward
r_HB_	1/4 per day	mean rate to transition from the normal ward to the ICU ward
r_HR_	1/14 per day	mean rate to transition from the normal ward to the removed state
r_BR_	1/20 per day	mean rate to transition from the ICU state to the removed state
p_IR_	fitted	fraction of infectious individuals (I) that enter the removed state (R) and are not hospitalized
p_HR_	fitted	fraction of hospitalized individuals in normal ward state (H) that are removed (R) and do not enter ICU state.
R_0_	fitted	R0-value, spreading dynamics

Values used for the model with three free parameters that are adjustable as new data emerges. Parameter values were taken from the Robert-Koch-Institute [25].

Aiming at predicting the occupancy of acute care, the input occupancies for the model are patient counts in the acute hospitals. Emergency reserves available in alternative medical facilities help keep up medical care during overburdening but are not an object of the regional coordination in tense but not overloaded periods.

On the day of prognosis the three free model parameters were fitted via the least squared error method to data from eight days prior to the prognosis day. Implicitly, this assumes that the trend for these parameters of the dynamics and people requiring attendance at hospitals remains unchanged during the prediction horizon. The model was then run from the day of prognosis with these parameters to forecast to seven days and give a trend line for 14 days in the future.

The fitting procedure was applied to each of the three hospital clusters individually. The sum of each of these regions then yields the state-wide prognosis for Saxony.

### Statistical methods for model validation

We validated our mathematical model for near-future regional bed capacity prognosis using two different approaches. At first, we scatter plot prognosis data against measured reality to visually guide the search for abnormalities in our prognosis across the orders of magnitude of the time series. An unbiased prediction across value ranges shows a symmetric cloud around the identity diagonal.

Secondly, we looked at the relative errors R(t) given by

$$R\left(t\right)=\frac{\left[forecast(t)-data(t)\right]}{data\left(t\right)}.$$

Persistently positive values as well as negative values of R(t) reveal systematic over- or underestimation and are thus an indicator of bias.

By treating the three clusters as an ensemble of statistically independent processes subject to the same time-dependent forcing of the pandemic, we get a picture of our model’s performance over time.

The distribution of relative errors summarizes the performance in a given time window T. The mean relative error R_mean_ and the standard deviation R_std_ of this distribution characterize the accuracy and the precision of the prediction. For an unbiased prediction, we expect R_mean_≈ 0. The predictions are only informative for small R_std_.

Since the infection dynamic is non-stationary and subject to sudden changes in the population’s behavior and the implementation of NPIs, we expected the performance to be time dependent. Thus, for a fair analysis of the prediction quality it is necessary to compute R_mean_ and R_std_ for different time windows T.

Finally, we computed R_mean_ and R_std_ for all of Saxony and for each individual regional cluster to evaluate the overall and regional performance.

## Results

In the following, we will present key figures as well as the model evaluation during the so-called second wave of infections in the federal state of Saxony. We briefly summarize the overall numbers of detected cases in Saxony and of occupancies in Saxonian hospitals, and present how the distribution of age, an important risk factor of hospitalization, has changed during the wave. This distribution provides an intuitive connection to the need of hospital beds, as the probability of hospitalization rises with age. The age distribution thus–if reported immediately—poses, in addition to the prognosis, an important source of information for medical management.

Finally, we will evaluate the performance of the model prediction on bed occupancy for Saxonian acute hospitals and the clusters by comparing our predicted and the true occupancies.

### Overview

By December 31, 2020, a total number of 138,549 individuals tested positive for SARS-CoV-2 since the beginning of the pandemic in Saxony (Table 2). Of these, the majority of 130,977 individuals were infected during the second wave from October 1 to December 31, 2020. The highest number of occupied beds in Saxony during that period was on December 29 with 3242 COVID-19 patients on normal ward and on December 31 with 521 patients on ICU. In Saxony, there were a total of 4,571 deceased COVID-19 patients by the end of 2020, including 4,327 in the last three months of the year. The central clinic coordination center in East-Saxony allocated 7,107 COVID-19 patients to the cluster’s clinics in 2020.

**Table 2:** The second COVID-19 wave in Saxony in numbers.

Oct. 1 –Dec. 31, 2020	Eastern Saxony	Western Saxony	Northern Saxony	Saxony
Incidence	58,167	42,872	29,938	130,977
(entire 2020)	(60,868)	(45,873)	(31,808)	(138,549)
Peak occupancy	1245	1477	578	3242
Normal ward	(22^nd^ December)	(29^th^ December)	(28^th^ December)	(29^th^ December)
Peak occupancy	217	215	98	521
ICU patients	(28^th^ December)	(31^st^ December)	(24^th^ December)	(31^st^ December)
Deceased	2,191	1,384	752	4,327
	(2,275)	(1,517)	(779)	(4,571)

Total numbers of incidences and deceased and peak numbers of occupancies on normal ward and ICU in Saxony and the clusters. Other than only acute hospitals for the prognosis, the numbers listed here count the overall occupancies in all regional facilities of medical inpatient care.

**Fig 4:**
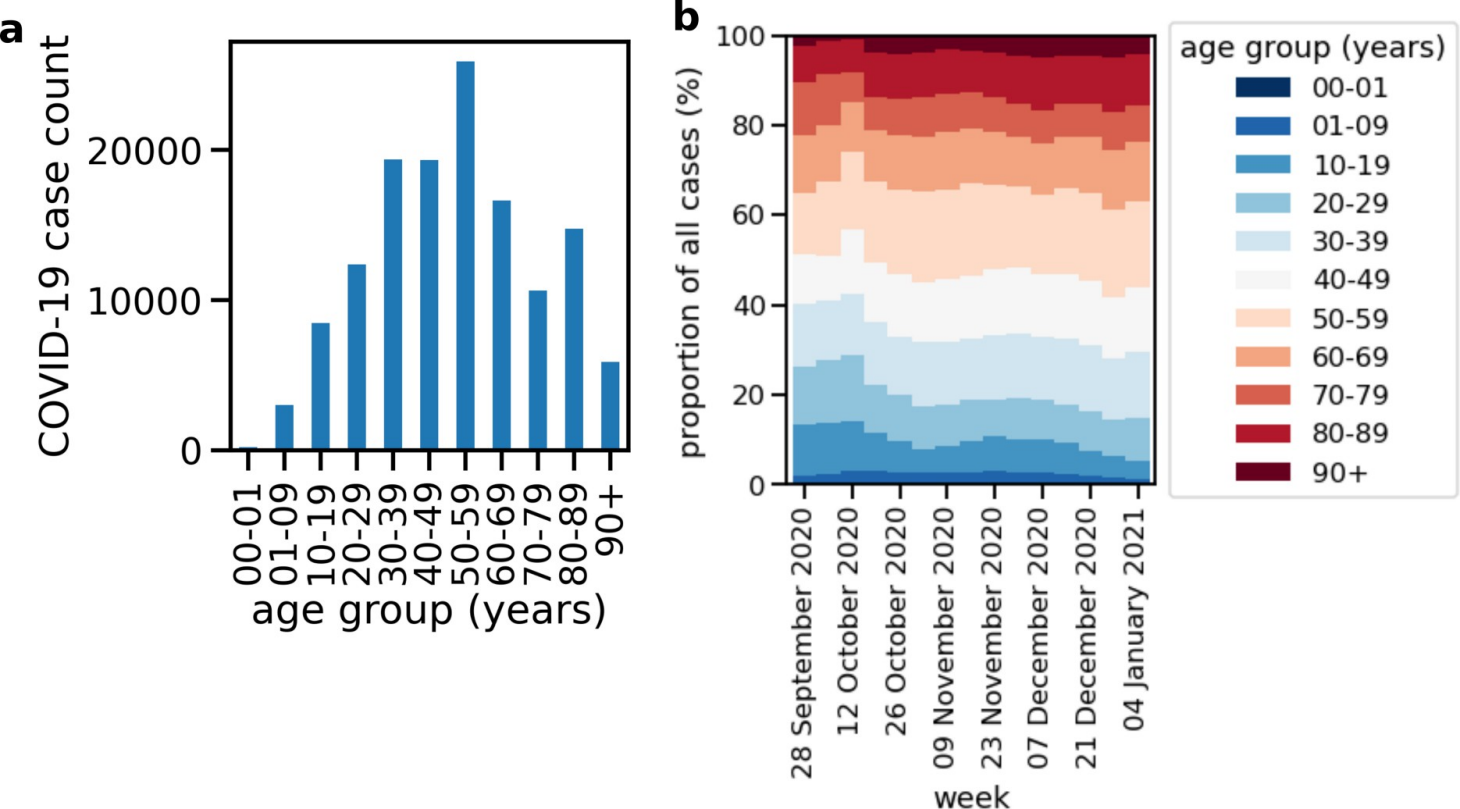
Age stratification of positively tested individuals in Saxony. (a) Age distribution of positively tested persons in Saxony between October 1 and December 31, 2020. (b) Distribution of COVID-19 affected age groups per week as a function of time.

Across all age groups more women than men were tested positive with prominent peaks in the age groups 50–59 and above 80 years, respectively (Fig 4A). In the very beginning of the second wave in late September 2020 younger age groups below 60 years were affected at a larger portion than the elderly (Fig 4B). The portion of incident cases in older people grew in October 2020. The distribution of positive tests among age groups then remained essentially constant over the course of the second wave until the end of the year 2020.

**Fig 5:**
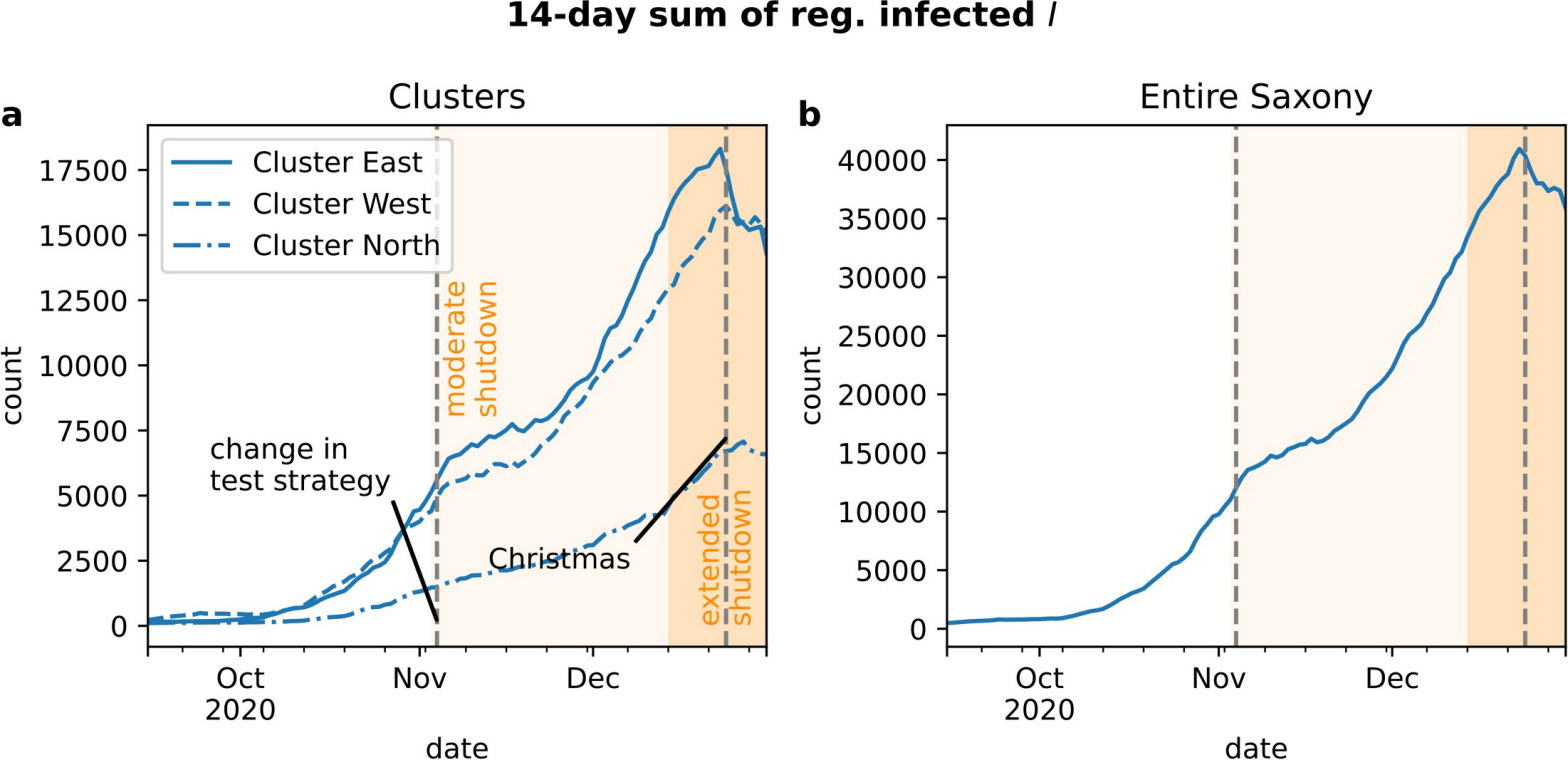
Course of the second COVID-19 wave in Saxony. Time series of 14-day sum of registered infected individuals. Data for (a) individual clusters and (b) all of Saxony shows dynamic effects caused by NPIs. The major difference between clusters is the amplitude; the overall picture is the same for each region and for entire Saxony.

The trajectory of registered individuals infected with SARS-CoV-2 in Saxony exhibited an exponential increase in October 2020, a slowing down and a plateau in November 2020 followed by a second almost linear increase in December 2020, a slowing down in late December 2020 and a rapid decrease before Christmas (see Fig 5).

### Model evaluation

The incorporated SEIR-model provides a seven-day estimate of the bed demand on ICU and normal ward in each of the three Saxon clusters. Since the qualitative infection dynamic was the same in all three clusters (i.e. the same shape and qualitative behavior) and since the resulting effects in our prognosis were analogous, we restricted our model evaluation to the cluster East Saxony and the whole state of Saxony. The performance evaluation of the two remaining clusters can be found in the S1 File accompanying this work.

**Fig 6:**
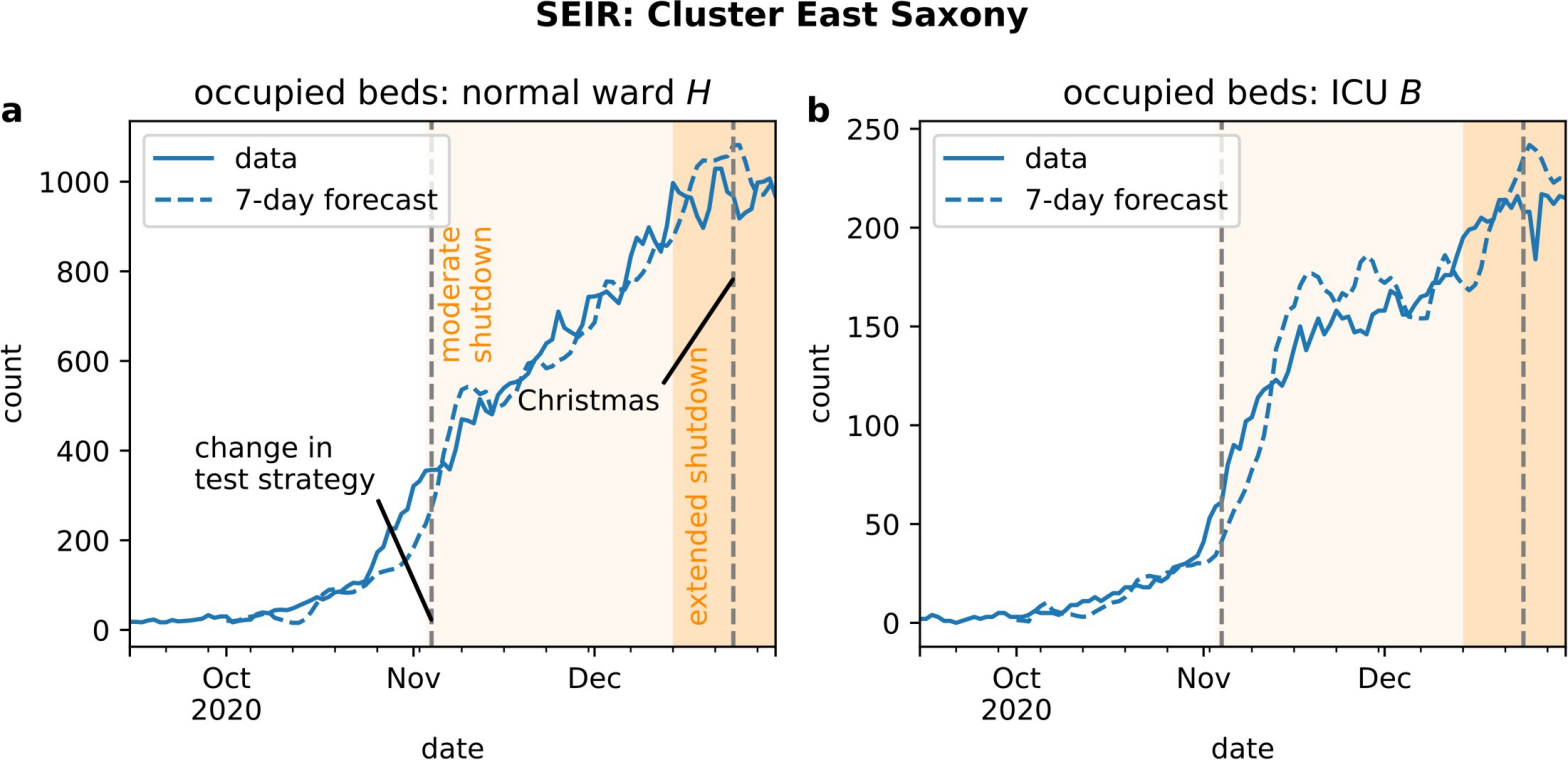
Bed demand in East Saxony during the second wave. Time series (solid lines) of (a) number of normal ward beds and (b) number of ICU beds occupied by COVID-19 patients show effects caused by NCIs, changes in test strategy and the Christmas holidays. The predictions (dashed lines) provided an accurate seven-day forecast.

**Fig 7:**
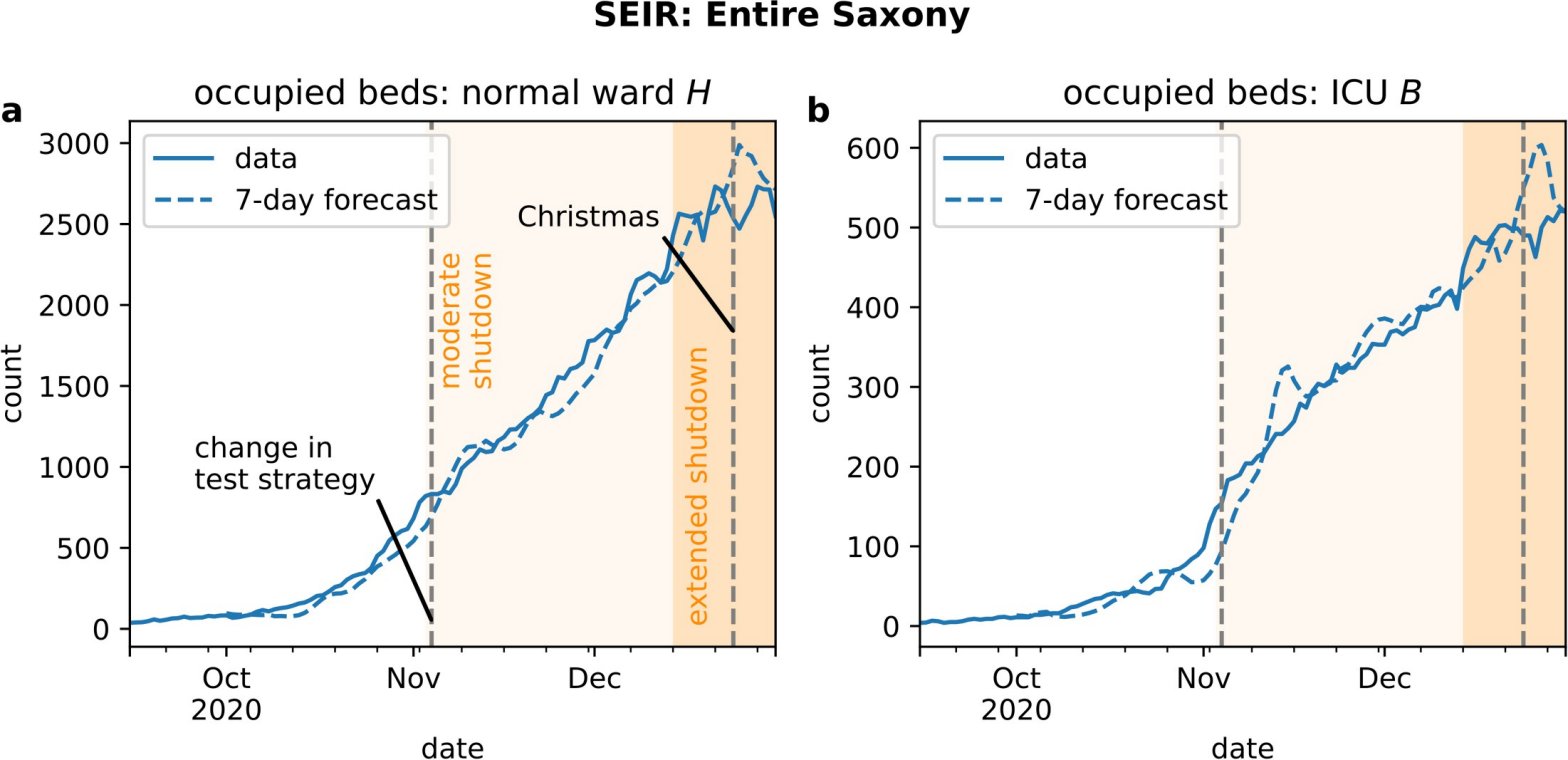
Total bed demand for Saxony during the second wave. Time series (solid lines) of (a) number of normal ward beds and (b) number of ICU beds occupied by COVID-19 patients are less noisy then single cluster time series and the prediction (dashed lines) more precise.

Our seven-day prognosis for bed demand in East Saxony reflected the behavior of the actual time series in general (Fig 6, dashed lines). The exponential increase in October, the slowing down and the plateau in November were all represented in the predictions. However, two deviations were noticeable. First, the model underestimated bed demands in normal ward at the end of October 2020 and in ICU in the beginning of November 2020 both for a duration of approximately ten days. Secondly, the predicted demand in ICU beds exhibited an overshoot in mid-November 2020 (see Fig 6A). The prognosis for entire Saxony appeared to be more accurate since the evolution of bed occupancy is visibly less noisy (Fig 7). The overshoot in predicted ICU bed demand was also visible here.

**Fig 8:**
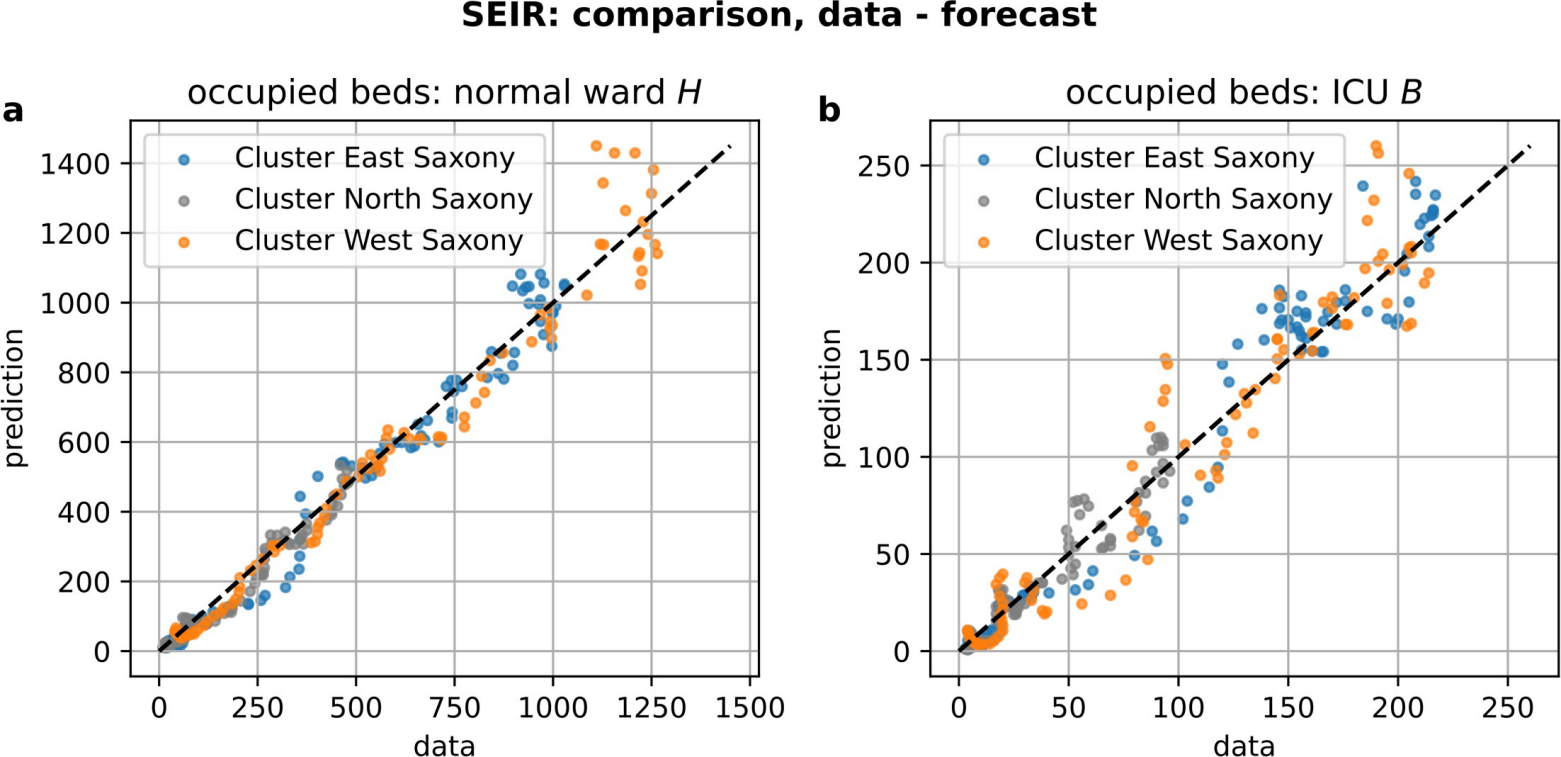
Scatter plot of actual data and predicted values. The plot shows a uniform corridor around the diagonal and displays no significant abnormalities.

The scatter plot revealed that the errors of the employed forecasting model form a uniform corridor and exhibited no visible biases for different ranges of predicted values (see Fig 8).

**Fig 9:**
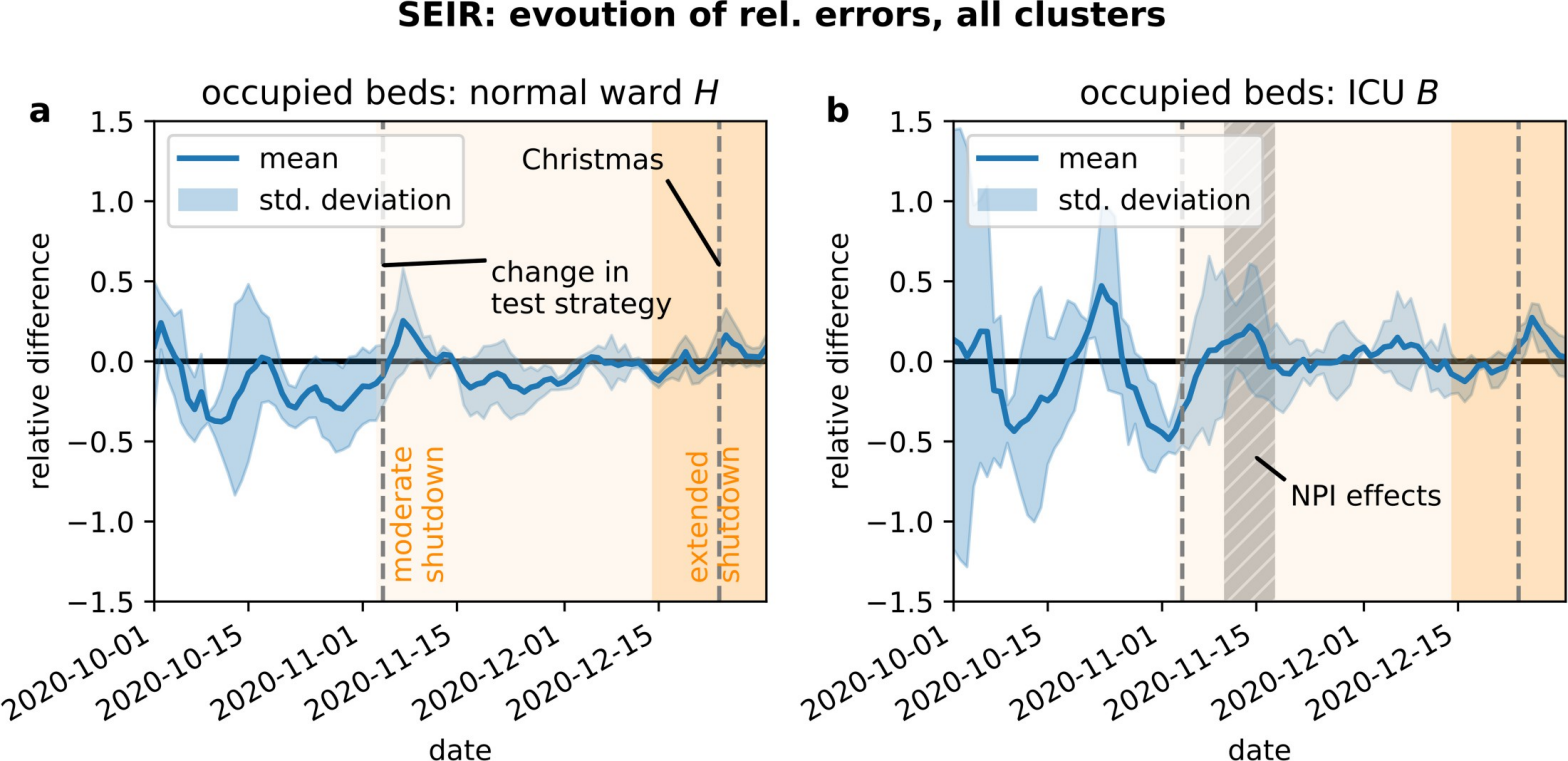
Time dependent distribution of relative errors among the three clusters. The quality of the prediction increases in the course of the second wave.

As we outlined in the Section Statistical Methods for Model Validation, we computed the time series of relative errors in bed demand for each of the three clusters and investigated their joint mean and standard deviation to assess the models performance over time as well as possible temporal biases (see Fig 9). The average relative errors in normal ward bed demand predictions among clusters showed a slight underestimation during October 2020. The amplitude of mean relative errors as well as the standard deviation of relative errors in normal ward prediction were decreasing continuously from October to December 2020. The average relative error in ICU bed demand prediction showed no such bias, but in contrast, large amplitudes in October 2020. Also, the corridor of standard deviation shows large deviations from the true value in October 2020. However, these large amplitudes in both mean and standard deviation were strongly reduced starting November 2020. In the beginning of November 2020, the mean relative error among clusters showed a correlated overestimation in ICU bed demand. The amplitude of the average relative errors among clusters decreased over time for both normal ward and ICU.

Motivated by these different performances during different time windows, we computed the mean and the standard deviation of relative errors for three periods. First, (A) during the complete three months October, November and December 2020, then (B) starting from the exertion of shutdown measures at November 3, 2020 until end of December 2020, and finally (C) in the time window starting at November 19, 2020, right after the overestimation period of ICU bed demand predictions, until end of December 2020. Table 3 shows the result of this calculation.

**Table 3:** Prediction quality of model.

Relative Differences		Entire Saxony	Cluster East Saxony
(Model—Data)/Data		(Mean +/- Std. Dev.)	(Mean +/- Std. Dev.)
(A) Oct 1—Dec 31, 2020	Normal ward	-0.08 +/- 0.21	-0.08 +/- 0.21
	ICU	-0.02 +/- 0.38	-0.05 +/- 0.29
(B) Nov 3—Dec 31, 2020	Normal ward	-0.07 +/- 0.14	0.00 +/- 0.11
	ICU	0.02 +/- 0.22	0.01 +/- 0.17
(C) Nov 19—Dec 31, 2020	Normal ward	-0.04 +/- 0.12	-0.00 +/- 0.08
	ICU	0.03 +/- 0.15	0.05 +/- 0.11

Accuracy (mean relative error) and precision (standard deviation of relative errors) of seven-day prognosis for entire Saxony and the East cluster for normal ward and ICU bed demand for different time windows (with and without periods of critical system changes). (For North and West Saxony, see S1 File).

In every time window the prognosis errors (standard deviation of relative errors) for the cluster East Saxony was lower than for the entire state of Saxony. We found that our model yields an expected error of 21% in normal bed occupancy and 38% in ICU bed occupancy for all of Saxony between October 1, 2020, and December 31, 2020, however, less than 15% during the critical phase of the second wave in November and December 2020.

## Discussion

In principle, Surveillance of SARS-CoV-2 spreading allows inferring the emergence of near-future COVID-19 cases by mathematical modeling. Numerous well-established epidemiological studies state a broad theoretical background for the modeling of infection dynamics. A common approach to this question are SEIR-type compartment models [21] along with stochastic Markov [26] or agent-based models [27]. Facing the resulting challenges for the health care system requires an extension of these models by dynamics that reflect inpatient flows. In case of SEIR-models, a direct solution to this task is adding compartments (Fig 3) for the different status of patients in medical treatment as proposed by [20, 23, 26].

The limitations of our current study manifest mainly in the available data to faithfully predict unknown model parameters. For instance, given the age-dependent severity of the disease course for a population requires exact knowledge of each individual’s exact age at each point in time. To circumvent this problem we implicitly modelled this parameter dependence by adjusting free parameters over time to reflect severity and current mean age of the infected individuals without actually requiring to know these exact variables. This goes in line with the principle of parsimony (also known as Occam’s razor) to only use a minimal number of variables to model a process. In addition, SEIR-models are a simplified view of complex disease dynamics and only have limited validity under stochastic effects such a low number of infected individuals. In order to reduce parameters even further, we omitted to model the transition between the infected and the ICU compartment because of its negligible role in Saxony during this study’s time frame. However, despite its limitations, as we showed, our approach suffices to predict hospital bed demand and guide the hospital planning process especially under a surge in Corona virus cases.

Accurate prognoses of near-future hospital capacity demands require an appropriate modeling of both infection dynamics and patient stays in hospital treatment. Predictions of infection dynamics help quantifying future patient influx into the healthcare system. Super-spreading events such as outbreaks in care homes, institutions of common education or collective accommodation or during other gatherings of larger groups of people notably accelerate infection spreading by a high number of affected people at the same time. Mathematical models cannot predict the occurrence of such events due to the complexity of human behavior. Prognoses can nonetheless account for and properly incorporate such observations if a detailed surveillance and reporting of detected cases and hotspots is provided. For the estimation of hospital capacity demands at specific points in time additional knowledge about the outflow of patients is necessary. This in turn requires information about the current occupancy of normal wards and intensive care units as well as the patient flow rates between model compartments.

The course of the pandemic during the spreading phase since the beginning of October 2020 is subject to non-pharmaceutical interventions and other changes of human behavior. Interventions systematically change both the dynamics of the pandemic and hospital admission and hence the requirements on mathematical models for health care demands. Until the end of 2020 the impact of the worldwide vaccination program due to immunization was still negligible in Germany. The exponential growth of reported infections was suspended by the implementation of light shutdown measures during November (Fig 4). The again rising numbers in early December 2020 were met with stronger shutdown regulations by mid of December 2020. During the Christmas period, surveillance data became less reliable due to a number of national holidays. The characteristics of the infections (Fig 5) are unexpected from a standard SEIR-curve and were mutually caused by NPIs (moderate shutdown: November 3, 2020, extended shutdown: December 16, 2020), changes in the national test strategy (only testing people with symptoms and proven contacts with infected individuals after November 4) and less testing and registrations during the Christmas holidays. The three clusters showed the same infection dynamics since the NPIs came into effect Saxony-wide at the same time.

The specific dynamical features were also visible to a lesser extent in the number of hospital beds occupied by COVID-19 patients on normal ward and ICU ward (see Fig 7).

Legal regulation affected the amount of hospital bed capacities in Germany due to financial reimbursements and interruption of elective medical treatment. During the Christmas holidays and on weekends the burden for the Saxon clinics in treating COVID-19 patients was even higher because of reduced outpatient medical treatment by general practitioners. For the future, the cooperation of outpatient and clinical treatment of COVID-19 patients has to be intensified to avoid unnecessary admissions and crowding in clinics.

The overall pandemic situation is obviously non-stationary, in the sense that the dynamics of infections and hence hospital admissions underlie different forces and characteristics during different temporal periods. Our model evaluation shows periods of better and worse forecast accuracy for hospital bed demands. The origin of the underestimation of bed demands (Fig 8) seems to be a sudden increase in bed occupancy in normal ward after October 23, 2020, and its delayed counterpart in ICU on October 31, 2020, which is not captured by the model and for which we do not have any explanation so far. Since this increase is not reflected by the incidences (compare Fig 4), this sudden jump in bed occupancy could hint towards an unforeseen shift in hospitalization risk, e.g. caused by a sudden change in the age distribution of infected individuals.

Furthermore, decreased outpatient treatment capacity and local out-breaks in rest homes may explain this haziness.

The temporal overestimation of ICU beds is most probably caused by the introduction of NPIs in the beginning of November. This is indicated by the start of the overestimation period around November 12, 2020, i.e. nine days after NPIs were introduced, which is the delay expected for such effects to be noticeable at ICU (four days of incubation period and six days between onset of disease and hospitalization at ICU [28–32]). Also, the overestimation period lasted about one week, i.e. the length of the prediction horizon, after which the model self-corrected and showed a more realistic trend which was visible in regional as well as Saxon wide data.

Taken together, in particular, these points illustrate that in the critical phase of increasing daily infection numbers, the predicted numbers of hospital demand reflect not the real development but rather a scenario that would have occurred if no counter measures had been introduced. As a remark, the prognosis for the entire state of Saxony is smoother (cp. Figs 6 and 7) since unavoidable stochastic errors in individual clusters average out.

The free parameters R_0_ (controls infections), p_IR_ and p_HR_ (relative flux sizes to removed compartment R from normal ward H and ICU ward B) in our modeling allow for a flexible and quick adjustment of our prognoses to the aforementioned systematic changes in the daily live application. The non-stationary setting of variable data influx (holidays, test strategy), systematic changes (NPIs, hospital admission policies) and prognoses that possibly induce interventions itself makes an overall model evaluation difficult. Considering the entire period of increasing incidence from October until December 2020, the error of our prognoses was 21% nonetheless. During November and December 2020, the critical phase of the high healthcare capacity demands and still ongoing growth of case numbers after the implementation of moderate NPIs, we achieved a prognosis precision of 15% at best. Considering sudden surges in hospitalization numbers caused by correlated infections in residence homes as well as planned German-wide redistribution of patients [17], this is an overall satisfying result compared to less flexible modeling approaches. Simple linear or exponential extrapolations obey a tendency of critical under- or overestimation during systematic changes. The precision in bed demand predictions was even better for East Saxony alone with 11% standard deviation in relative errors. Predictions in North Saxony experienced increased difficulty simply because of less demand in hospital beds that resulted in increased relative variability of the data. We openly communicate the prediction uncertainty of our modeling to all stakeholders such as politicians and the clinic coordination centers. Additionally, we assess the validity of our prognoses on a daily basis by interpreting the results in the current context of possible expected or unexpected system changes due to NPIs or influencing factors like school holidays. This close interaction between mathematical modeling and decision making substantiates both our opportunity of providing reliable predictions and the trust of practical decision makers.

The utilization of the DISPENSE tool by the central clinic coordination centers was essential to avoid crowding and secure medical treatment in Saxony during the Christmas holidays at the peak of the second wave. Because the tool predicted exhausted treatment capacities within two weeks, the central COVID-coordination in Saxony decided on December 18 to launch LEVEL III of the aforementioned graduated distribution scheme and allocated COVID-19 beds nationwide in Germany. Because of large transportation distances in Germany, weather dependent flight conditions (rescue helicopter) and high logistical demands, these additional two weeks were valuable and helped to secure free hospital beds at any time in Saxony. According to this successful disaster management, the Saxonian concept of patient distribution located at university hospitals and based on close collaboration between political and clinical decision makers, data providers and mathematical modeling is a “best-practice” example among the German healthcare system.

## Conclusions

The DISPENSE project in the German Federal State of Saxony put a regional data-based patient steering system into practice during the COVID-19 pandemic. The online tool comprises a comprehensive quantification and visualization of the regional pandemic situation. A strong cooperation between the inpatient coordination centers, the data scientists in charge of the prognosis and the medical informatics department crucially strengthens the Saxon health care system by integrating the activities into each individual player’s work routine.

The resulting dashboard visually integrates data that would be otherwise disperse. The end-users and decision-makers have key information at their fingertip to adjust their course of actions to, for example, coordinate management teams to plan political social distancing interventions as in the case of the state ministry and to provide additional hospital resources as in the case of hospital overburdening.

The present case study brings together theoretical modeling of bed capacity demands and real bed occupancy data of the second pandemic wave in Saxony. Experiences from the daily live application of the model prognoses propose some improvements of the forecasting procedure such as adjustments to unexpected behavior in both the spreading and bed dynamics.

Modeling the course of expected hospital capacity demands during a pandemic benefits hospitals with more balanced workloads and resource demands.

## Supporting information

S1 File (PDF)
