# Supplementary material for: Regional responsibility and coordination of appropriate inpatient care capacities for patients with COVID-19 – the German DISPENSE model
Source: PLoS One. 2022 Jan 27;17(1):e0262491. doi: 10.1371/journal.pone.0262491 (PMC8794159; doi:10.1371/journal.pone.0262491)
Supplement: S1 File — (PDF) [file pone.0262491.s001.pdf]

Supplement Information: Regional responsibility and coordination of appropriate inpatient care capacities for patients with COVID-19 – The German DISPENSE model

Benedict J. Lünsmann[1$*], Katja Polotzek[1$], Christian Kleber[2], Richard Gebler[3], Veronika Bierbaum[1], Felix Walther[1,4], Fabian Baum[1], Kathleen Juncken[5], Christoph Forkert[1], Toni Lange[1], Hanns-Christoph Held[6], Andreas Mogwitz[7], Robin R. Weidemann[7], Martin Sedlmayr[3], Nicole Lakowa[5], Sebastian N. Stehr[6], Michael Albrecht[7], Jens Karschau[1%], Jochen Schmitt[1%]

[1] Center for Evidence-based Healthcare, University Hospital Dresden and Medical Faculty Carl Gustav Carus, TU Dresden, Dresden, Germany
[2] University Center of Orthopaedic, Trauma and Plastic Surgery, University Hospital Carl Gustav Carus, Dresden, Germany
[3] Institute for Medical Informatics and Biometry, University Hospital Dresden and Medical Faculty Carl Gustav Carus, TU Dresden, Dresden, Germany
[4] Quality and Medical Risk Management, University Hospital Carl Gustav Carus Dresden, Dresden, Germany
[5] Clinic for Infectious Diseases and Tropical Medicine, Klinikum Chemnitz, Chemnitz, Germany
[6] Department of Anesthesia and Critical Care Medicine, Leipzig University Hospital, Leipzig, Germany
[7] University Hospital Carl Gustav Carus Dresden, Dresden, Germany

* Corresponding author
Email: benedict.luensmann@tu-dresden.de

[$] These authors contributed equally to this work.
[%] These authors contributed equally to this work.

Supplement

Relative Differences (Model - Data)/Data	Cluster West Saxony (Mean +/- Std. Dev.)	Cluster North Saxony (Mean +/- Std. Dev.)
Normal ward (Oct 1 - Dec 31)	-0.06 +/- 0.17	-0.09 +/- 0.25
ICU (Oct 1 - Dec 31)	0.04 +/- 0.47	-0.04 +/- 0.35
Normal ward (Nov 3 - Dec 31)	-0.02 +/- 0.1	-0.05 +/- 0.20
ICU (Nov 3 - Dec 31)	0.01 +/- 0.22	0.04 +/- 0.25
Normal ward (Nov 19 - Dec 31)	-0.02 +/- 0.11	-0.09 +/- 0.15
ICU (Nov 19 - Dec 31)	0.02 +/- 0.13	0.01 +/- 0.19

Suppl. Tab 1. Prediction quality of model (with and without periods of critical system changes) in West and North Saxony.

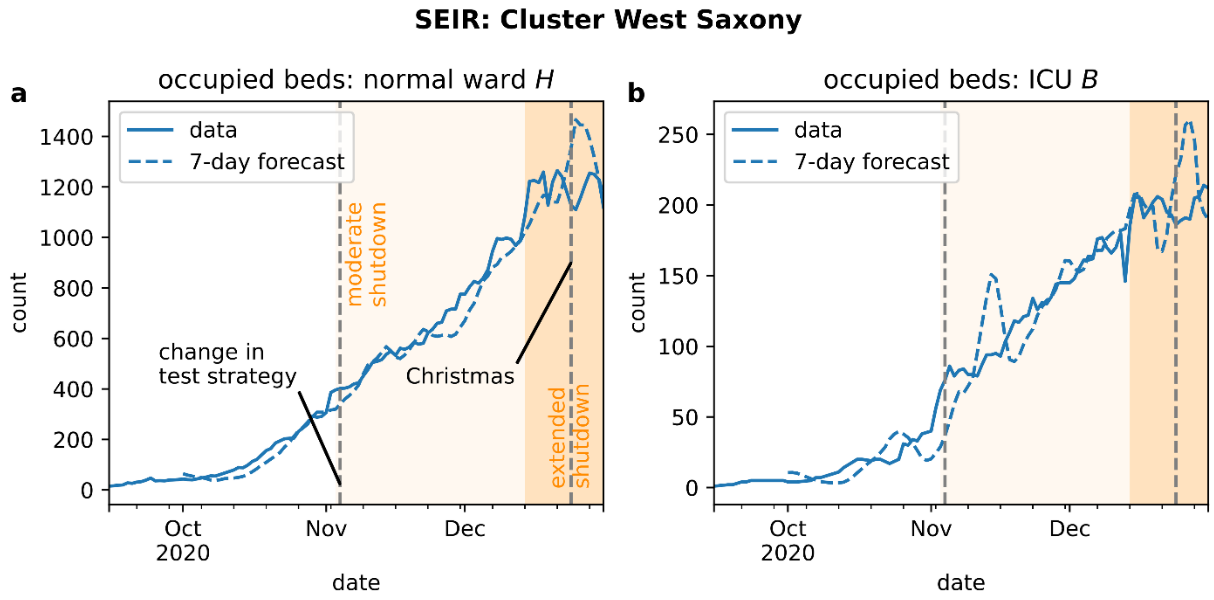

Suppl. Fig 1. Demand of hospital beds in normal ward and ICU in Western Saxony.

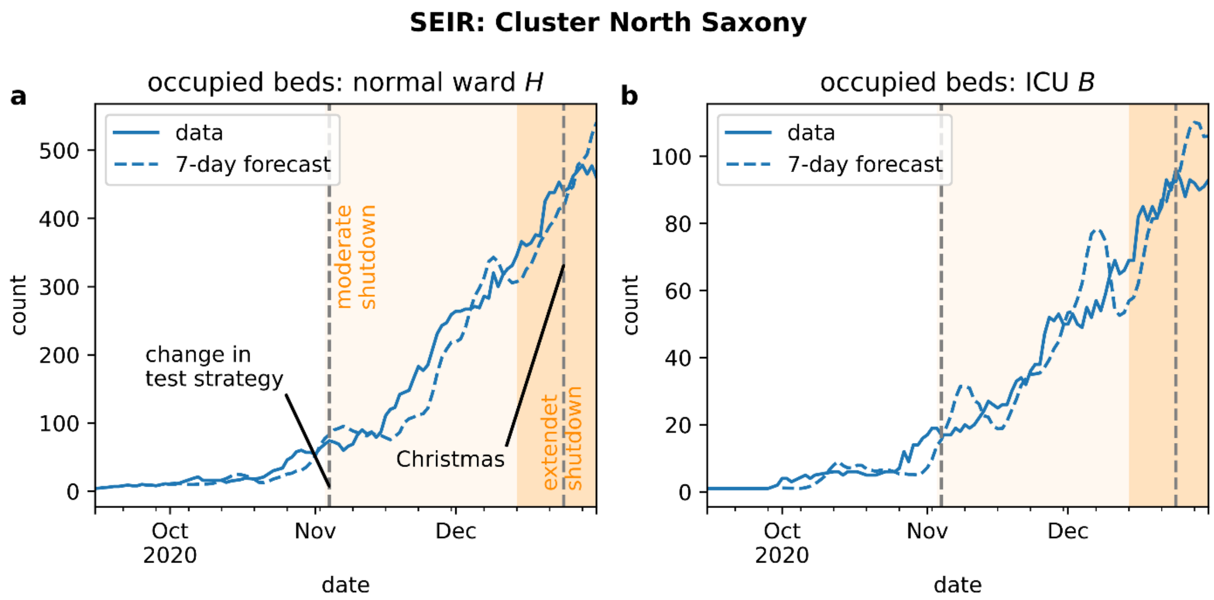

Suppl. Fig 2. Demand of hospital beds in normal ward and ICU in Northern Saxony. Reality (full lines) and seven-day prognosis (dashed).

Model equations of SEIR model with states similar to [33].

$$\frac{dS(t)}{dt} = -\frac{r_{SE}S(t)E(t)}{N}$$

$$\frac{dE(t)}{dt} = \frac{r_{SE}S(t)I(t)}{N} - r_{EI}E(t)$$

$$\frac{dH(t)}{dt} = p_{IH}r_{IH}I(t) - p_{HR}r_{HR}H(t) - (1 - p_{HR})r_{HB}H(t)$$

$$\frac{dB(t)}{dt} = p_{HB}r_{HB}H(t) - p_{BR}r_{BR}B(t)$$

$$\frac{dR(t)}{dt} = p_{IR}r_{IR}I(t) + p_{HR}r_{HR}H(t) + p_{BR}r_{BR}B(t)$$

$$r_{SE} = R_0 r_{IR}$$

Susceptible population S(t) , exposed population E(t), infectious population I(t), hospitalized population (normal ward) H(t), hospitalized population (ICU ward) B(t), removed population R(t), N = 4 M. Parameters: See Tab 1 in the main text.
